# Phase I Study of the Pharmacodynamics and Safety of Sodium Zirconium Cyclosilicate in Healthy Chinese Adults

**DOI:** 10.1002/cpdd.1055

**Published:** 2022-01-08

**Authors:** Tommy Cheung, Fang Sun, June Zhao, Yulin Qin, Mats Någård

**Affiliations:** ^1^ Department of Medicine The University of Hong Kong, Hong Kong, HKSAR China; ^2^ AstraZeneca Shanghai China; ^3^ AstraZeneca Wilmington Delaware USA; ^4^ Formerly AstraZeneca R&D China Shanghai China; ^5^ Clinical Pharmacology and Safety Sciences AstraZeneca R&D Gaithersburg Maryland USA

**Keywords:** Chinese, hyperkalemia, phase I, potassium, sodium zirconium cyclosilicate

## Abstract

Sodium zirconium cyclosilicate (SZC) is an effective potassium binder for patients with hyperkalemia. This single‐center, open‐label, phase I study (NCT03283267) characterized the pharmacodynamics and safety of SZC in Chinese individuals. Twenty‐two healthy Chinese adults (mean age, 33.5 years) randomized 1:1 received daily oral SZC 5 or 10 g for 4 days, following 4 days on a low‐sodium, high‐potassium diet (continued throughout the study). End points were mean change from baseline in 24‐hour urinary potassium (primary) and sodium excretion, and serum potassium concentration. Urinary potassium excretion significantly decreased with SZC 5 g (mean change [mmol], –13.0; *P* < .001) and 10 g (–15.4; *P* < .001). Although urinary sodium excretion decreased significantly with SZC 5 g (–11.5; *P* = .030), there was no significant change with SZC 10 g (–5.1; *P* = .299). Serum potassium concentrations decreased significantly with SZC 5 g (–0.14; *P* = .031) and 10 g (–0.20; *P* = .002). All treatment‐emergent adverse events were mild, and none were considered causally related to SZC. Over 4 days, the pharmacodynamics and safety of SZC were consistent in healthy Chinese adults with global studies and patients of Japanese ethnicity.

Hyperkalemia is a common and potentially serious medical condition. The disruption of potassium homeostasis can result from a number of possible factors, including excessive potassium intake, impaired potassium excretion due to renal insufficiency, or exposure to potassium‐sparing medications, such as renin‐angiotensin‐aldosterone system inhibitors and nonsteroidal anti‐inflammatory drugs.^1^, ^2^, ^3^ Hyperkalemia is thus frequently observed in patients with cardiovascular disease, heart failure (HF), chronic kidney disease (CKD), and diabetes, and is associated with significant long‐term economic burden and high rates of mortality.^1^, ^2^, ^4^, ^5^

The etiology and causal factors of hyperkalemia have been well characterized over >50 years of clinical research.^6^, ^7^, ^8^ Yet only in the previous decade have treatment options for hyperkalemia advanced from dialysis and the cation‐exchange resin sodium polystyrene sulfonate (SPS).^9^ Currently available treatment options for hyperkalemia include SPS and the oral potassium binders patiromer and sodium zirconium cyclosilicate (SZC).^10^, ^11^ SPS is an ion exchange resin that works by exchanging sodium for potassium in the colon. Although SPS has a positive benefit‐risk profile, colonic necrosis and other rare but serious gastrointestinal adverse reactions have been reported.^12^, ^13^ Patiromer is a nonabsorbable cation exchange polymer that binds potassium in the gastrointestinal tract and is approved for the treatment of hyperkalemia in adults in the United States and the European Union. Randomized controlled trials in hyperkalemic patients with HF or CKD have shown that patiromer can effectively lower serum potassium concentrations and maintain normokalemia.^11^, ^14^, ^15^, ^16^, ^17^ The most common adverse events (AEs) associated with patiromer administration were constipation, hypomagnesemia, diarrhea, nausea, abdominal discomfort, and flatulence.^11^, ^14^, ^16^, ^17^ In addition, patiromer can also bind certain charged medications in the gastrointestinal tract, such as ciprofloxacin, levothyroxine, warfarin, and metformin, and decrease their gastrointestinal absorption.^18^ As a precautionary measure, administration of patiromer is recommended at least 3 hours apart from other oral medications.

SZC is an orally administered, inorganic, nonabsorbed, highly selective potassium binder that is approved for the treatment of hyperkalemia in adult patients in the United States, European Union, and more recently in China. SZC selectively exchanges bound hydrogen and sodium ions for potassium through a specific interaction driven by cation charge and size of the nonhydrated cation.^10^, ^19^ As a result, SZC increases fecal potassium excretion through binding of potassium in the gastrointestinal tract, thereby reducing free potassium concentrations in the gastrointestinal tract and lowering serum potassium concentrations.^10^, ^19^ In 4 phase III clinical trials evaluating SZC in mainly White patients with hyperkalemia, most of whom had CKD (including those undergoing hemodialysis), HF, or diabetes, SZC not only reduced mean serum potassium concentrations to normal levels within 48 hours of treatment but also maintained normokalemia when compared with placebo.^20^, ^21^, ^22^, ^23^, ^24^ The most common AEs reported in patients treated with SZC were hypokalemia and edema.^20^ Among Japanese patients with hyperkalemia, SZC also provided rapid attainment of normokalemia, with no new safety concerns and favorable tolerability throughout a year of exposure in phase II/III clinical trials.^25^, ^26^

The effect of SZC on the excretion of sodium and potassium in healthy American adults receiving a standardized diet was evaluated in a recent phase I study.^27^ The results confirmed that treatment with SZC did not significantly affect urinary or fecal sodium excretion. SZC decreased serum and urinary potassium concentrations by increasing fecal potassium excretion. SZC was well tolerated, with no clinically meaningful changes in physical or laboratory parameters.^27^

To support the regulatory approval of SZC in China and further evaluate the effects of SZC on sodium homeostasis, the phase I study (NCT03283267) reported here evaluated the pharmacodynamics and safety of SZC in healthy Chinese adults.

## Participants and Methods

### Study Design

This was a single‐center, open‐label, phase I pharmacodynamic and safety study in healthy Chinese adults residing in Hong Kong who received a standardized, low‐sodium, high‐potassium diet. The study was conducted between October 24, 2017, and November 23, 2017. The primary objective of this study was to evaluate the effect of SZC on the urinary excretion of potassium in healthy Chinese adults maintained on a standardized diet, designed to eliminate potential confounders such as variabilities in dietary sodium and potassium intake. Secondary objectives included examining the effects of SZC on urinary sodium excretion, serum potassium concentrations, and serum concentrations of other electrolytes, and the safety and tolerability of SZC.

**Figure 1:**
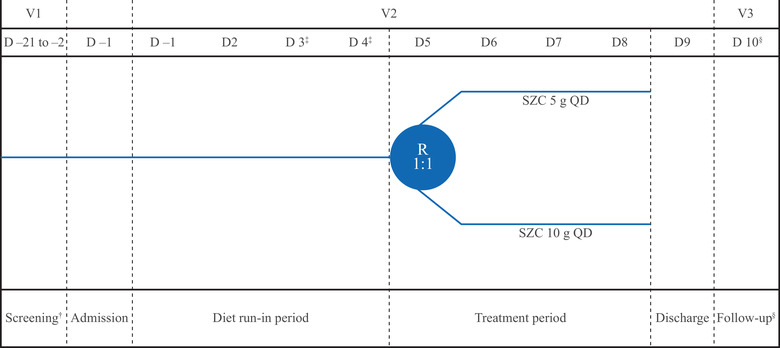
Study design. ^†^The screening visit occurred within 21 days of the 4‐day diet run‐in period. ^‡^Study days 3 and 4 were the baseline period. SZC was administered from day 5 to day 8. ^§^The follow‐up visit occurred 2 to 5 days after the last dose on day 8. D, day; QD, once daily; R, randomization; SZC, sodium zirconium cyclosilicate; V, visit.

The study participants were admitted to the phase I Clinical Trials Centre, Queen Mary Hospital, Hong Kong, China, on day –1 and remained there until discharge on day 9. Following a 4‐day run‐in period in which participants began the standardized diet (days 1 to 4), participants were randomized 1:1 to receive once‐daily oral SZC 5 or 10‐g powder suspended in water for 4 days (days 5‐8) (Figure 1). The standardized diet contained 40 (±10%) mmol/day (920 mg [±10%] per day) of sodium and 128 (±10%) mmol/day (4992 mg [±10%] per day) of potassium. A low‐sodium and high‐potassium diet was chosen to maximize the ability to detect any changes in urinary sodium excretion. It was hypothesized that this diet and the 4‐day run‐in period would deplete sodium and the high potassium content would maximize the amount of sodium released from SZC. Participants continued this diet until discharge on day 9.

Baseline urinary potassium and sodium excretion was determined by collecting 2 spot urine samples 24 hours apart on days 3 and 4 (baseline period), starting at 8 am or the same time on both days. Urinary electrolyte excretion during SZC administration was determined by collecting 2 samples 24 hours apart on days 7 and 8, each starting at the same time on both days, before breakfast. Total volume and total time of collection were measured to ensure completeness of 24‐hour urine samples.

### Eligibility

Healthy Chinese male and female adults (aged 18‐55 years) residing in Hong Kong were eligible for inclusion in the study. Participants had to be willing to consume food and beverages according to the standardized daily diet defined above, provided by the research center. Participants were excluded if they had clinically significant abnormalities from physical examination, laboratory tests, electrocardiogram (ECG), or vital signs at screening and admission; were positive for HIV or hepatitis B or C virus; had current and/or history of alcohol or drug abuse; or were receiving concomitant medications of vitamins, dietary supplements, and herbal preparations within 2 weeks before day 1.

The study protocol was approved by the independent Institutional Review Board of the University of Hong Kong/Hospital Authority Hong Kong West Cluster, and the study was conducted in accordance with regulations governing clinical trials, including the Declaration of Helsinki and the International Council for Harmonisation/Good Clinical Practice. Applicable regulatory requirements and the AstraZeneca policy on bioethics were also followed. All participants were required to provide informed consent before participating in the study. Participants were permitted to withdraw or discontinue from the study for any reason.

### Outcomes

The primary end point was mean change from baseline in urinary potassium excretion following SZC administration. This was evaluated by comparing the average daily urinary potassium excretion on days 3 and 4 with the average daily urinary potassium excretion on days 7 and 8. Secondary end points included mean change from baseline in urinary sodium excretion (average daily urinary sodium excretion on days 3 and 4 compared with average daily urinary sodium excretion on days 7 and 8) and mean change in serum potassium from baseline (average concentrations on days 3, 4, and 5, before administering the study drug, compared with average concentrations on days 7 and 8). Safety and tolerability were also evaluated, including occurrence of AEs, classified according to the terminology of Medical Dictionary for Regulatory Activities version 20.0. All subjects were followed up by telephone interview on days 10 to 13 to record any postdischarge AEs and serious AEs (SAEs). Treatment‐emergent AEs (TEAEs) were defined as any event that started after the first dose of treatment and was not present at baseline, or any event representing an exacerbation of a condition present at baseline. Potential effects on other serum electrolytes (calcium, magnesium, sodium, phosphate, and bicarbonate) and blood urea nitrogen (BUN) were also evaluated. All participants also underwent standard safety evaluations, including vital signs, ECG, physical examination, and clinical laboratory parameters.

### Statistical Analysis

A study sample size of 10 participants per treatment group was determined to be sufficient for assessing the mean change from baseline in urinary potassium excretion. The effect size was calculated as the mean divided by the standard deviation. The study had 80% power to detect an effect size of 1 using a 2‐sided paired *t* test at a significance level of 0.05 for urinary potassium excretion.

The primary variable was change in urinary potassium excretion. Daily results within each period were averaged to form the individual baseline (days 3 and 4) and mean results after SZC treatment (days 7 and 8). Mean results by period and changes from baseline were summarized using descriptive statistics for each dose group. The null hypothesis that the mean change is 0 was tested using a 1‐sample, 2‐sided paired *t* test for the primary end point. This was performed separately for each dose group (SZC 5 and 10 g/day). The results of all statistical analyses were presented using a 95% CI and 2‐sided *P* value, unless otherwise stated. A 2‐sided *P* value of <.05 was considered statistically significant. No adjustments were made for multiplicity.

The secondary end points (urinary sodium excretion and serum potassium concentrations) were analyzed in the same manner. Safety variables and other laboratory assessments were summarized descriptively.

## Results

### Participants

Of 28 enrolled Chinese adults, 6 failed to meet the eligibility criteria and did not enter the diet run‐in period. A total of 22 individuals (13 men and 9 women) were randomized 1:1 to receive SZC 5 g or 10 g/day. The 2 groups were well balanced with respect to baseline and demographic factors, medical history, and concomitant medications, and mean age across the groups was 33.5 years (Table 1). All 22 randomized study participants were included in the efficacy and safety analyses.

**Table 1:** Baseline and Demographic Characteristics

Characteristic	SZC 5 g QD (N = 11)	SZC 10 g QD (N = 11)	Overall (N = 22)
Age, y, mean (SD)	30.0 (6.2)	37.0 (11.5)	33.5 (9.7)
Sex, n (%)			
Male	7 (63.6)	6 (54.5)	13 (59.1)
Female	4 (36.4)	5 (45.5)	9 (40.9)
Body mass index, kg/m^2^, mean (SD)	22.5 (4.0)	22.3 (2.5)	22.4 (3.3)
Any medical history, n (%)^a^	4 (36.4)	5 (45.5)	9 (40.9)
Any surgical history, n (%)^b^	4 (36.4)	3 (27.3)	7 (31.8)
Any concomitant medication, n (%)^c^	5 (45.5)	3 (27.3)	8 (36.4)
Antipropulsives (loperamide)	2 (18.2)	2 (18.2)	4 (18.2)
Anilides (paracetamol)	2 (18.2)	0 (0.0)	2 (9.1)
Antacid (with antiflatulents)	1 (9.1)	0 (0.0)	1 (4.5)
H2‐receptor antagonist (famotidine)	1 (9.1)	0 (0.0)	1 (4.5)
Emollient/protective (mucopolysaccharide polysulfuric acid ester)	0 (0.0)	1 (9.1)	1 (4.5)

QD, once daily; SD, standard deviation; SZC, sodium zirconium cyclosilicate.

^a^ No more than 1 participant in each group had a history of the following: epidermal nevus, growth hormone deficiency, peptic ulcer, impacted tooth, cellulitis, epididymitis, corneal abrasion, foot deformity, uterine leiomyoma, neuralgic amyotrophy, adjustment disorder, dysmenorrhea, allergic rhinitis, dermal cyst, ingrown nail, or rosacea.

^b^ No more than 1 participant in each group had undergone the following procedure: laser therapy, maxillofacial operation, scrotal exploration, wedge resection toenail, or wisdom tooth removal.

^c^ Includes medications that began before admission (study day –1) and were ongoing after admission.

### Urinary Potassium Excretion

**Figure 2:**
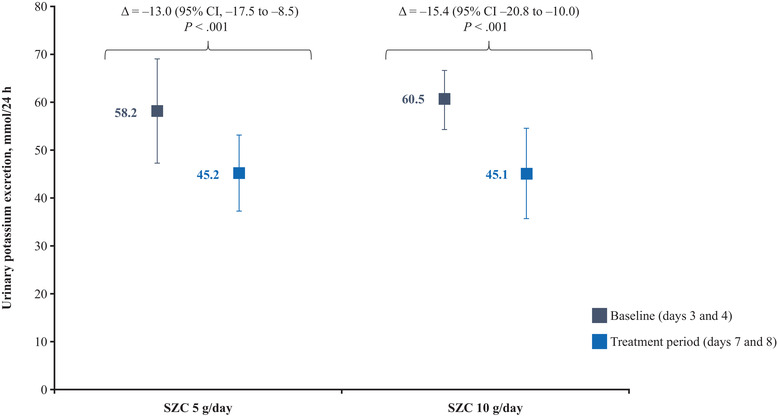
Urinary potassium excretion at baseline and following treatment with SZC. Data points represent mean ± standard deviation. SZC, sodium zirconium cyclosilicate.

There was a statistically significant decrease in urinary potassium excretion after SZC administration compared with baseline for both doses tested (Figure 2). In the group treated with SZC 5 g/day, mean urinary potassium excretion decreased from 58.2 mmol/24 h at baseline to 45.2 mmol/24 h after receiving SZC (a change of –13.0 mmol/24 h [95% CI, –17.5 to –8.5]; *P* < .001). In the group treated with SZC 10 g/day, mean urinary potassium excretion decreased from 60.5 mmol/24 h at baseline to 45.1 mmol/24 h (a change of –15.4 mmol/24 h [95% CI, –20.8 to –10.0]; *P* < .001).

### Urinary Sodium Excretion and Serum Potassium Concentrations

**Figure 3:**
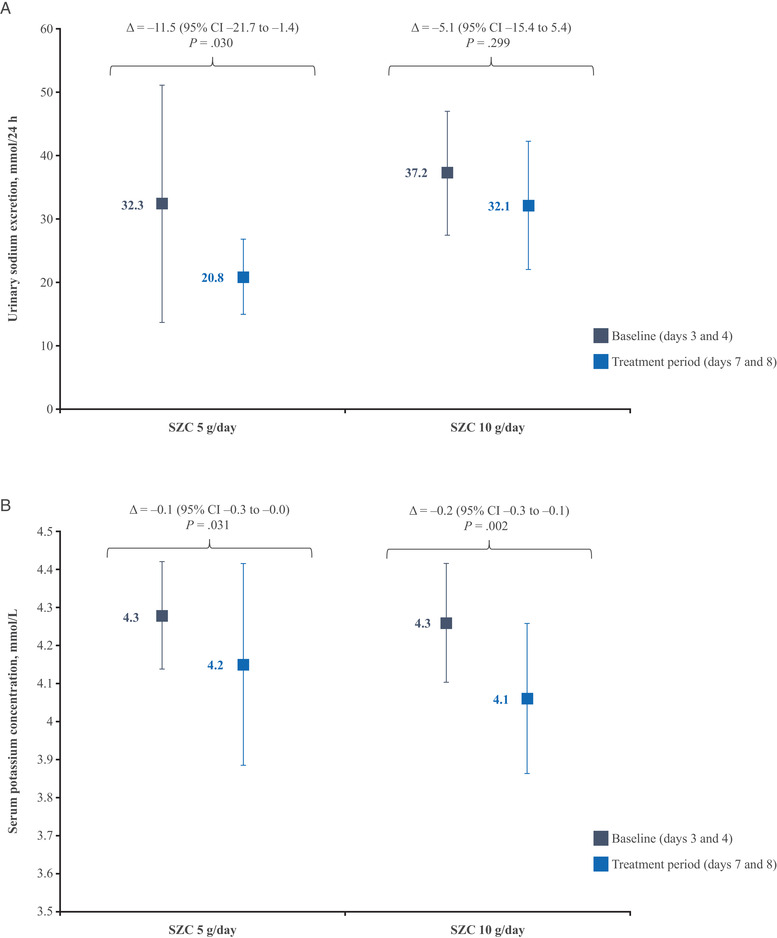
Urinary sodium excretion (A) and serum potassium concentrations (B) following treatment with SZC. Data points represent mean ± standard deviation. SZC, sodium zirconium cyclosilicate.

In the group treated with SZC 5 g/day, mean urinary sodium excretion was 32.3 mmol/24 h at baseline, and 20.8 mmol/24 h after receiving SZC (Figure 3A). This represented a statistically significant decrease (–11.5 mmol/L/24 h [95% CI, –21.7 to –1.4]; *P* = .030). However, there was no statistically significant change in mean urinary sodium excretion (37.2 mmol/24 h at baseline and 32.1 mmol/24 h following treatment; a change of –5.1 mmol/L/24 h [95% CI, –15.4 to 5.4]; *P* = .299; Figure 3A) among participants treated with SZC 10 g/day.

There was a statistically significant decrease in serum potassium concentrations after SZC administration compared with baseline for both doses tested (Figure 3B). Mean serum potassium concentrations decreased from 4.28 mmol/L at baseline to 4.15 mmol/L following SZC administration (a change of –0.14 mmol/L [95% CI, –0.26 to –0.02]; *P* = .031) among participants receiving SZC 5 g/day. In the group treated with SZC 10 g/day, mean serum potassium concentrations decreased from 4.26 mmol/L at baseline to 4.06 mmol/L (a change of –0.20 mmol/L [95% CI, –0.31 to –0.09]; *P* = .002).

### Other Serum Electrolytes and BUN

There were no clinically meaningful changes from baseline in serum bicarbonate, calcium, magnesium, phosphate, sodium concentrations, or BUN during the study period in both treatment groups (Table 2).

**Table 2:** Other Serum Electrolytes, Blood Urea Nitrogen, and Serum Creatinine at Baseline and Day 9, Following Treatment With SZC 5 g or 10 g Daily

Serum Electrolytes^a^	SZC 5 g QD (N = 11)	SZC 10 g QD (N = 11)
Serum bicarbonate, mmol/L		
Baseline	20.90 (2.88)	20.29 (0.71)
Day 9	21.38 (1.88)	21.68 (1.62)
Change from baseline, % (SD)	3.51 (12.45)	6.88 (7.55)
Calcium, mg/dL
Baseline	9.54 (0.32)	9.30 (0.40)
Day 9	9.58 (0.28)	9.42 (0.24)
Change from baseline, % (SD)	0.48 (2.85)	1.22 (2.47)
Magnesium, mg/dL		
Baseline	2.50 (0.12)	2.53 (0.15)
Day 9	2.38 (0.10)	2.38 (0.15)
Change from baseline, % (SD)	–4.41 (4.16)	–5.65 (2.88)
Phosphate, mg/dL		
Baseline	4.00 (0.40)	3.72 (0.43)
Day 9	4.74 (0.56)	4.43 (0.50)
Change from baseline, % (SD)	19.11 (12.07)	19.27 (6.96)
Sodium, mmol/L		
Baseline	137.3 (1.19)	136.5 (1.81)
Day 9	138.2 (1.54)	138.5 (1.21)
Change from baseline, % (SD)	0.67 (1.05)	1.47 (0.89)
Blood urea nitrogen, mg/dL		
Baseline	19.83 (2.83)	21.65 (3.14)
Day 9	17.78 (3.05)	18.68 (2.38)
Change from baseline, % (SD)	–10.51 (6.87)	–13.02 (9.67)
Serum creatinine, mg/dL		
Baseline	0.83 (0.15)	0.81 (0.19)
Day 9	0.88 (0.15)	0.87 (0.20)
Change from baseline, % (SD)	5.84 (9.53)	8.30 (8.59)

QD, once daily; SD, standard deviation; SZC, sodium zirconium cyclosilicate.

Baseline was defined as the last result obtained before the start of study treatment.

^a^ Values shown are mean (SD) unless otherwise stated.

### Safety

All 22 randomized participants received 4 doses of SZC over 4 days, giving a total exposure of 44 days for each SZC dose. During the overall study period, 16 participants (72.7%) experienced an AE: 9 participants received SZC 5 g/day and 7 received SZC 10 g/day. Twelve participants (54.5%) reported an AE during the diet run‐in period: 7 (63.6%) in the SZC 5 g/day group, and 5 (45.5%) in the SZC 10 g/day group. AEs reported were considered most likely due to the change in diet, with the most commonly reported AEs being diarrhea and dyspepsia, occurring in 8 (36.4%) and 4 (18.2%) participants, respectively (Table 3).

**Table 3:** AEs Reported During the Diet Run‐in Phase and the SZC Treatment Period

Adverse Event	SZC 5 g QD (N = 11)	SZC 10 g QD (N = 11)	Overall (N = 22)
Diet run‐in phase			
Any AE	7 (63.6)	5 (45.5)	12 (54.5)
Diarrhea	4 (46.4)	4 (36.4)	8 (36.4)
Dyspepsia	2 (18.2)	2 (18.2)	4 (18.2)
Headache	2 (18.2)	1 (9.1)	3 (13.6)
Abdominal pain	1 (9.1)	1 (9.1)	2 (9.1)
Back pain	1 (9.1)	0 (0)	1 (4.5)
Eczema	1 (9.1)	0 (0)	1 (4.5)
Urticaria	1 (9.1)	0 (0)	1 (4.5)
SZC treatment period			
Any AE	4 (36.4)	6 (54.5)	10 (45.5)
Dyspepsia	2 (18.2)	4 (36.4)	6 (27.3)
Diarrhea	1 (9.1)	2 (18.2)	3 (13.6)
Headache	1 (9.1)	1 (9.1)	2 (9.1)
Subcutaneous hemorrhage	0 (0)	1 (9.1)	1 (4.5)

AE, adverse event; QD, once daily; SZC, sodium zirconium cyclosilicate.

Values are n (%).

The diet run‐in phase was from day 1 to day 4. Participants did not receive any SZC treatment during the diet run‐in phase. The SZC treatment period was from day 5 to day 8.

In total, 10 participants (45.5%) reported TEAEs during the SZC treatment period: 4 (36.4%) in the SZC 5 g/day group and 6 (54.5%) in the SZC 10 g/day group. As in the diet run‐in phase, these were mainly dyspepsia (2 [18.2%] and 4 [36.4%] participants in the SZC 5 g/day and SZC 10 g/day groups, respectively) and diarrhea (1 [9.1%] and 2 [18.2%] participants, respectively) (Table 3). One participant in each group experienced headache, and subcutaneous hemorrhage was reported by 1 participant in the SZC 10 g/day group. All TEAEs were mild in intensity, and none were considered causally related to SZC. There were no AEs of edema or hypokalemia reported among all study participants (all receiving the standardized low‐sodium, high‐potassium diet). There were no SAEs or AEs leading to study discontinuation reported during the study.

There were no clinically meaningful changes from baseline in hematology, serum chemistry parameters, ECG measurements, physical examination (weight), or vital signs (pulse, blood pressure, and body temperature) in either treatment group (data not shown).

## Discussion

In this phase I study, both SZC 5 and 10 g daily doses significantly reduced urinary potassium excretion and serum potassium concentrations in healthy Chinese adults receiving a standardized low‐sodium, high‐potassium diet. These results support the mechanism of action of SZC, which selectively binds potassium in the intestinal tract, leading to the fecal excretion of serum potassium and a systemic reduction in potassium concentrations.^19^ Furthermore, these findings are comparable with those achieved in a phase I study in healthy American adults, which used a similar dosing regimen and standardized low‐sodium, high‐potassium diet.^27^ In the healthy volunteer study in the United States, the mean (standard deviation) change in urinary potassium excretion from baseline to study days 7 and 8 was –9.7 (18.0) mmol/24 h with SZC 5 g, and –21.0 (21.0) mmol/24 h with SZC 10 g, the latter being statistically significant (*P* < .05).^27^

Although statistically significant, observed reductions from baseline in serum potassium concentration with SZC were small (up to –0.20 mmol/L). Of note, these reductions were observed in a population of healthy Chinese adults without hyperkalemia who received a high‐potassium diet to reduce the risks of hypokalemia due to the potassium‐lowering mechanism of SZC, and are consistent with those observed among healthy American adults receiving a similar high‐potassium diet.^27^ Indeed, the ability of SZC to provide significant and clinically meaningful reductions in serum potassium concentration and maintain normokalemia among patients with hyperkalemia has been demonstrated for up to 12 months in phase II/III clinical trials,^20^, ^21^, ^22^, ^23^, ^24^ including among Asian patients.^25^, ^26^

The primary focus of phase II and III clinical trials of SZC to date has been on reductions in serum potassium levels in patients with hyperkalemia and comorbidities. However, several of these studies have also reported an absence of clinically relevant effects on serum sodium concentrations.^20^, ^22^, ^24^, ^28^ In the present study, participants in both the SZC 5 g/day and SZC 10 g/day dose groups experienced a decrease in urinary sodium excretion. This decrease was significant for the SZC 5 g/day dose, but not for the SZC 10 g/day dose. Similarly, in the US phase I study, urinary sodium excretion was decreased numerically but not significantly in either dose group (–0.9 [25.85] mmol/24 h with SZC 5 g/day, and –5.5 [13.90] mmol/24 h with SZC 10 g/day).^27^ Although these findings are suggestive that SZC does not release sodium for absorption and excretion, similar to the US phase I study, it is possible that participants were still adapting to the low‐sodium diet when SZC administration was started, and therefore reduction of dietary sodium masked any increase in sodium by SZC.^27^ However, in our study, there were no events of edema or clinically meaningful changes in blood pressure and body weight suggestive of sodium retention with SZC.

Among the exploratory outcomes assessed, serum bicarbonate increased, and BUN decreased following SZC, both in a dose‐dependent manner. These observations are consistent with those from phase III SZC studies of patients with hyperkalemia.^29^ The observed increase in serum bicarbonate is likely due to SZC binding of gastrointestinal ammonium, while decreases in serum urea are associated with SZC effects on serum bicarbonate.^29^ Indeed, these findings in healthy volunteers are of note as maintaining serum bicarbonate ≥22 mmol/L is a major goal for the treatment of CKD.

Treatment with SZC was well tolerated. While TEAEs occurred in 10 (45.5%) participants, overall they were mild in intensity and not considered to be related to SZC. In fact, AEs occurring during SZC treatment were very similar to those reported during the diet run‐in phase and mainly consisted of diarrhea and dyspepsia. Furthermore, despite the reduction in serum and urinary potassium concentrations, no incidences of hypokalemia or edema were observed, and there were no SAEs, AEs leading to study drug discontinuation, or deaths during the study.

Potential limitations of this study include the use of SZC in conjunction with a standardized low‐sodium, high‐potassium diet. This diet was designed to maximize the detection of changes in sodium and potassium concentrations after SZC administration, and this does not reflect normal physiological environments or diets. A systematic review of 24‐hour urinary sodium and potassium excretion in China found that the average dietary intake of sodium exceeded approximately double the recommended level across all age groups, while potassium intake was less than half that generally advised.^30^ It is also possible that the 4‐day diet run‐in phase in our study was not long enough to allow sodium levels to reach full equilibrium before SZC administration. As a phase I study among healthy volunteers, comparative efficacy and safety relative to other treatment options for hyperkalemia were not evaluated. Finally, only healthy individuals were enrolled in this phase I study. Participants did not have hyperkalemia or hyperkalemia‐associated comorbidities and were therefore not taking concomitant medications commonly administered to such patients. Thus, the potential effects of concomitant medications on electrolyte concentrations could not be assessed.

## Conclusions

In conclusion, this phase I study in healthy Chinese adults indicated that SZC reduces urinary potassium excretion and serum potassium concentrations, with no increase in urinary sodium excretion over 4 days of treatment. These observations were consistent with the known mechanism of action for SZC. The pharmacodynamic and safety profiles of SZC in Chinese subjects were similar to those observed in the global population.

## Conflicts of Interest

F.S., J.Z., and M.N. are employees of AstraZeneca. Y.Q. was an employee of AstraZeneca at the time of the study. M.N. is a shareholder of AstraZeneca. T.C. received grants and nonfinancial support from AstraZeneca at the time of the study.

## Funding

This study was supported by AstraZeneca. The sponsor was involved in the study design, collection, analysis, and interpretation of data, as well as data checking of information provided in the article. However, ultimate responsibility for opinions, conclusions, and data interpretation lies with the authors.

## Author Contributions

M.N., Y.Q., F.S., and J.Z. contributed to the study design. T.C. collected the data for the study. M.N., Y.Q., F.S., and J.Z. performed the data analysis and interpretation. All authors critically reviewed the manuscript, approved the final version, and accept accountability for the overall work.

## Data Sharing

Data underlying the findings described in this article may be obtained in accordance with AstraZeneca's data sharing policy described at https://astrazenecagroup‐dt.pharmacm.com/DT/Home.
